# How digital transformation curb greenwashing: Insights from fraud risk factor theory

**DOI:** 10.1371/journal.pone.0339282

**Published:** 2026-02-02

**Authors:** Jiajun Xu, Rui Li

**Affiliations:** School of Finance and Economics, Jimei University, Xiamen, China; Namik Kemal University: Tekirdag Namik Kemal Universitesi, TÜRKIYE

## Abstract

This study investigates how digital transformation influences corporate greenwashing and promotes genuine sustainable development, drawing on the framework of fraud risk factors. Based on panel data from Chinese publicly listed companies between 2009 and 2022, a two-way fixed effects model is employed, with endogeneity addressed through difference-in-differences and instrumental variable techniques. The results show that digital transformation significantly curbs greenwashing by mitigating motivations, reducing opportunities, and enhancing exposure. The effect is stronger for growth- or mature-stage enterprises, non-myopic firms, and regions with low regulatory intensity and high environmental awareness. Furthermore, a double-threshold effect is identified, with the inhibitory role of digital transformation becoming more significant at intermediate and advanced stages. Importantly, digital transformation reduces greenwashing without compromising firms’ financial or sustainable performance. These results provide actionable insights for businesses and policymakers in curbing greenwashing and advancing sustainable development.

## 1. Introduction

While some companies actively fulfill their environmental responsibilities in advancing green development, others adopt superficial or deceptive practices to capitalize on public attention—a phenomenon known as greenwashing [1–3]. Greenwashing involves low-cost methods such as false advertising, exaggeration of environmental achievements, or selective disclosure of environmental information, which help firms gain brand value, investment, and policy incentives [4–6]. However, such practices undermine market fairness, erode public trust [7,8], and hinder genuine green transformation, while also posing risks to global environmental governance and sustainable development [9]. Against this backdrop, understanding how to curb greenwashing is of great research significance. With rising environmental awareness among consumers and investors [4,10], companies are under pressure to demonstrate authenticity in their sustainability efforts, which calls for effective mechanisms to restrain deceptive practices.

Amidst the rapid growth of the digital economy, technological advancements such as big data, artificial intelligence (AI), blockchain, and cloud computing have become the foundation of digital transformation. These technologies not only reshape production and management processes but also enhance transparency and traceability of environmental practices, making digital transformation an indispensable pathway for sustainable development. Existing studies, however, mainly focus on economic performance [11–14], with limited exploration of its role in shaping corporate social responsibility (CSR) and environmental, social, and governance (ESG) outcomes. Current literature tends to emphasize external drivers—regulations, market demand, and societal expectations [15–17]—or isolated internal factors such as governance structures and executive incentives [18–20], lacking an integrated theoretical framework to explain how digital transformation curbs greenwashing.

This study contributes to the literature in three important ways. First, it introduces the fraud risk factor theory to develop a systematic framework showing that digital transformation suppresses greenwashing by weakening motivations, reducing opportunities, and increasing exposure. This enriches our understanding of how technological advancement constrains deceptive behavior and strengthens environmental credibility. Second, it empirically investigates the heterogeneous effects of digital transformation using panel data of Chinese listed firms from 2009 to 2022, considering different types of digital technologies, firm characteristics, and regional contexts. Third, it reveals a nonlinear “double-threshold” effect, with stronger suppression of greenwashing at intermediate and advanced stages of digitalization, and demonstrates that digital transformation achieves these benefits without harming firms’ financial or sustainability performance. By clarifying the mechanisms, heterogeneity, and economic consequences, this study highlights both the theoretical and practical importance of digital transformation in steering corporations toward genuine sustainability and offers new insights for managers and policymakers.

## 2. Theoretical mechanism

Building on previous analyses of greenwashing, this study frames corporate greenwashing as a form of fraud, particularly when it involves deliberately misleading consumers or investors about the environmental attributes, practices, or performance of its products or operations. Greenwashing behavior may include exaggerating the effectiveness of charitable efforts, intentionally concealing socially harmful practices, or presenting a misleading pro-social image through marketing tactics, while failing to take corresponding social responsibility actions. This behavior aligns with the core elements of the fraud risk factor theory, which involve moral factors, motivational factors, opportunity factors, the likelihood of exposure, and the extent of punishment faced. According to the fraud risk factor theory [21], corporate greenwashing behavior results from the interplay of multiple factors. Within this theoretical framework, the motivations of the actors are primarily determined by moral and motivational factors, while the opportunity to implement these behaviors provides the necessary conditions. Additionally, the likelihood of exposure and the severity of potential punishments serve as situational factors that exert a moderating effect on corporate greenwashing activities. Notably, under the assumption of economic rationality, corporate decision-making processes do not inherently incorporate moral considerations. Therefore, this analysis excludes the impact of moral factors on greenwashing behavior, focusing instead on the critical role of digital transformation in this context. Moreover, since digital transformation cannot alter the penalties imposed by governments and markets following the exposure of greenwashing activities, this study will examine the influence of digital transformation on fraud risk factors from the perspectives of motivation, opportunity, and exposure rate.

### 2.1. Weakening greenwashing motivation

The motivation for corporate greenwashing can be summarized as two main goals: reducing compliance costs and increasing economic gains. This section explores how digital transformation can weaken these motivations by addressing both aspects.

Firstly, as environmental regulations tighten and public awareness rises, companies face increasing compliance pressure, prompting them to seek cost-effective solutions [22]. Failure to meet environmental standards can result in fines and production restrictions, leading some companies to resort to greenwashing as a strategy to project compliance and reduce costs [23,24]. By superficially implementing green initiatives, companies can simplify compliance processes and gain a competitive edge [25]. Digital transformation, through technologies like big data and Internet of Things (IoT), enhances real-time monitoring and efficiency in green innovation, reducing compliance costs and, consequently, the inclination towards greenwashing [26].

On the other hand, economic incentives drive greenwashing as companies seek to attract sustainable investors and improve market competitiveness by showcasing environmental commitments [9]. However, exposure risks can lead to reputational damage. Digital transformation helps mitigate financial constraints by optimizing operations, reducing costs, and enhancing transparency, thus lessening the drive for greenwashing by improving financial conditions and market positioning [27].

In summary, companies engage in greenwashing to reduce compliance costs and increase economic gains. While this strategy carries exposure risks, digital transformation offers a solution by improving green innovation efficiency and financial transparency, thereby reducing the motivation for greenwashing and fostering genuine sustainable practices.

### 2.2. Reducing greenwashing opportunities

According to the analysis of the fraud risk factor theory, opportunity factors refer to the conditions under which a corporate may engage in greenwashing. These conditions are constrained by external regulations and internal control capabilities. The absence of legal standards and the inadequacies in regulatory frameworks provide external opportunities for greenwashing, while weak internal control systems and accountability mechanisms make such unethical behavior feasible. Given that digital transformation is unlikely to directly impact external regulation, this discussion will focus on how digital transformation affects internal control capabilities, thereby reducing opportunities for corporate greenwashing.

The digital transformation enables businesses to achieve real-time data collection and analysis, enhancing their internal information management capabilities. By establishing a centralized data platform and monitoring the implementation of environmental measures in real-time, a deeper understanding of business operations is achieved, alongside increased supervision of management and employees [28]. Real-time monitoring and data analysis tools in the digital environment bolster the effectiveness of internal auditing, enabling the timely detection of non-compliant behavior and identification of underlying issues [29]. As the digital transformation progresses, businesses achieve automation and standardization of their workflows, reducing opportunities for human intervention and minimizing the likelihood of greenwashing practices [30]. Furthermore, this automation improves production efficiency, allowing businesses to pursue sustainable development while simultaneously considering economic interests [31,32].

In summary, digital transformation improves internal information management through real-time data collection and analysis, establishing centralized data platforms, enhancing business understanding and oversight, and reducing opportunities for greenwashing. It also increases the effectiveness of internal audits by enabling dynamic auditing and data-driven analysis to quickly identify and correct non-compliant behavior. Furthermore, the automation and standardization of business processes reduce human intervention, thereby decreasing the likelihood of greenwashing. Consequently, digital transformation enhances internal control capabilities, thereby reducing the opportunities for corporate greenwashing.

### 2.3. Increasing greenwashing exposure

According to the fraud risk factor theory, when the likelihood of exposing corporate greenwashing increases, companies face risks such as government penalties, reputational damage, and loss of market share, which can deter such behavior.

During digital transformation, companies are not only under the scrutiny of media and the public but also need to effectively signal to investors the validity of their strategies [33]. As noted by Ren, Zeng [34], stakeholder attention significantly increases towards companies undergoing transformation, compelling them to emphasize their environmental performance. This heightened scrutiny forces companies to present transparent and accurate information to mitigate risks associated with information asymmetry. As digital transformation progresses, the adoption of cutting-edge technologies like big data and blockchain makes tracking and recording activities easier, significantly enhancing internal transparency. Lv and Chen [35] found that media attention increases notably when companies actively pursue digital transformation, making self-serving behaviors more likely to be exposed. Such exposure not only elevates public oversight but also pressures managers to maintain high standards in environmental practices.

Digital transformation significantly enhances information fluidity, allowing stakeholders easier access to data about a corporate’s environmental performance and activities. This transparency reduces corporate control over information dissemination, making any misleading greenwashing actions quickly noticeable. Research indicates that technological advancements not only boost organizational transparency but also increase stakeholder visibility [36]. In this context, the media plays a crucial role in disseminating information, offering in-depth reports on corporate environmental performance. Any environmental shortcomings or violations, once exposed by the media, spread rapidly online, generating substantial public pressure that forces companies to acknowledge and correct the issues. In this process, the media not only acts as a reporter but also serves as a catalyst driving improvements in corporate environmental practices [37,38].

In conclusion, digital transformation significantly increases the exposure of greenwashing by enhancing corporate information transparency, reducing information asymmetry, and attracting greater media and public attention. This mechanism not only prompts companies to self-regulate in environmental matters but also requires them to act with greater caution and responsibility in their sustainability efforts. Facing public pressure and stakeholder scrutiny, companies must adapt openly and truthfully to the challenges and opportunities presented by digital transformation, contributing positively to sustainable development for society and the environment.

**Fig 1:**
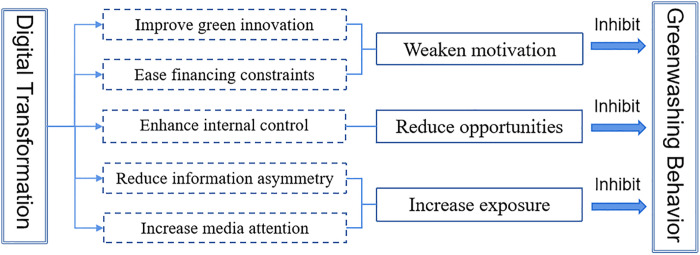
The influence mechanism of digital transformation on corporate greenwashing.

Overall, digital transformation hinders corporate greenwashing by weakening the motivation for greenwashing, reducing opportunities for greenwashing, and increasing the exposure rate of greenwashing, as illustrated in Fig 1.

## 3. Research design

### 3.1. Regression model

This study constructs a two-way fixed-effects model to explore the impact of digital transformation on corporates’ greenwash behavior, with the following baseline regression model.


$$GW_{f,t}=a_{1}+\beta_{1}\;Dig_{f,t}+\beta_{2}Z_{f,t}\;+\;\mu_{f}\;+\;\theta_{t}\;+\;\varepsilon_{f,t}$$ (1)

where f and t denote the corporate and the year respectively; $GW_{f,t}$ represents the degree of greenwashing of the corporate; $Dig_{f,t}$ is the degree of digital transformation of the corporate. $\beta_{1}\;$ is the impact coefficient of digital transformation on corporates’ greenwashing behavior, if it is significantly less than 0, it means that digital transformation effectively inhibits corporates’ greenwashing behavior. $Z_{f,t}$, $\mu_{f}$, $\theta_{t}$ and $\varepsilon_{f,t}$ denote control variables, individual fixed effects, time fixed effects and disturbance terms, respectively. Since both explanatory and interpreted variables are firm-level indicators, this study clusters standard errors to the firm level.

### 3.2. Variable definition

#### 3.2.1. Explanatory variable.

The explanatory variable in this study is the degree of digital transformation of corporates (*Dig*). Building upon the methodology proposed by Wu, Hu [39], Python web scraping techniques were used to extract keywords related to digital transformation from the annual reports of listed companies. These keywords were sourced from local government reports, the 14th Five-Year Plan for Digital Economy Development, and research reports from authoritative institutions. The keywords were divided into two levels: “underlying technological architecture” (including artificial intelligence, big data, cloud computing, and blockchain) and “technological application practices.” In total, there were more than 100 keywords. The degree of digitalization of corporates was measured by the frequency of these keywords, which was calculated by dividing the frequency of relevant keywords by the total word count of the annual reports. This yielded the digital transformation index (*Dig*) of corporates, which was then multiplied by 10,000. Detailed information on the construction of digital transformation can be found in the Appendix.

#### 3.2.2. Explained variable.

The explained variable in this study is the degree of greenwashing of corporates (*GW*), which measures the gap between companies’ claimed environmental responsibility and their actual performance using the disclosure-performance matching method. Building on previous research [40,41], the disclosure score of environmental information was measured using the environmental disclosure score from the Bloomberg ESG scoring system. The actual environmental performance was measured using the environmental performance score from the Huazheng ESG Index. The difference between the standardized scores of these two measures was used to quantify the degree of greenwashing of corporates.

#### 3.2.3. Control variable.

To more accurately estimate the relationship between digital transformation and greenwashing behavior and mitigate the potential biases caused by unobservable factors, this study introduces a series of control variables from both firm characteristics and corporate governance perspectives. Firm characteristics variables include firm size (*Size*), leverage ratio (*Lev*), proportion of fixed assets (*Fixed*), years of listing (*Listage*), and cash flow ratio (*Cashflow*). Corporate governance variables include board size (*Board*), dual roles of CEO and chairman (*Dual*), and proportion of independent directors (*Indep*). Additionally, in robustness checks, this study also controls for a range of regional factors that may affect greenwashing behavior, such as economic development level (*Pgdp*), population density (*Pop*), technological progress (*Tech*), and human capital (*Hum*). The detailed measurements of firm-level and regional-level control variables are presented in Table 1.

**Table 1:** Control variables.

Type	Variable	Symbole	Measurement
Firm-level	Firm size	*Size*	The logarithm of total assets at the end of the year
	Leverage ratio	*Lev*	The ratio of total liabilities to total assets
	Proportion of fixed assets	*Fixed*	The ratio of net fixed assets to total assets
	Years of listing	*Listage*	The logarithm of the difference between the current year and the year of listing
	Cash flow ratio	*Cashflow*	The ratio of net cash flow from operating activities to total assets at the end of the year
	Board size	*Board*	The logarithm of the number of current directors
	Dual roles	*Dual*	1 if the chairman and CEO are the same person, 0 otherwise
	Proportion of independent directors	*Indep*	The percentage of independent directors to total directors
Regional-level	Economic development level	*Pgdp*	The logarithm of actual per capita GDP in the prefecture-level city
	Population density	*Pop*	The ratio of the year-end population to the administrative area
	Technological progress	*Tech*	The ratio of internal R&D expenditure to regional GDP
	Human capital	*Hum*	The ratio of the population with at least a bachelor’s degree to the total population of the city

### 3.3 Sample selection and data sources

Considering that the data on environmental performance scores has been publicly available since 2009 and due to the availability of control variables, this study sets the sample period from 2009 to 2022. The following data processing steps were applied to the obtained raw data: (1) excluding ST, ST*, and PT companies within the sample period; (2) excluding samples with missing values for key variables; (3) excluding companies in the financial industry; (4) transforming non-ratio variables into logarithms to reduce heteroscedasticity and non-stationarity of the data; (5) imputing a small number of missing values, using the average growth rate method for initial and final years and linear interpolation for intermediate years. After these steps, a total of 1,403 listed companies with 13,079 observations were obtained. The disclosed data of listed companies used in this study are sourced from the China Securities Market and Accounting Research (CSMAR) database and the China Research Data Services (CNRDS) database. The environmental performance score data is sourced from the Wind database. Regional-level data is sourced from the annual China Statistical Yearbook and China City Statistical Yearbook. Descriptive statistics of the variables are shown in Table 2.

**Table 2:** Descriptive statistics.

Variable	Obs.	Mean	Std. dev	Min	Max
*GW*	13079	0.002	1.229	−4.879	6.515
*Dig*	13079	0.528	1.429	0.000	21.714
*Size*	13079	23.151	1.304	19.317	26.452
*Lev*	13079	0.479	0.199	0.027	0.908
*Fixed*	13079	0.228	0.177	0.002	0.769
*ListAge*	13079	2.430	0.737	0.000	3.401
*Cashflow*	13079	0.060	0.071	−0.222	0.283
*Board*	13079	2.180	0.202	1.609	2.708
*Dual*	13079	0.205	0.403	0.000	1.000
*Indep*	13079	37.511	5.548	25.000	60.000

## 4. Empirical results

### 4.1. Benchmark regression results

Table 3 illustrates the impact of digital transformation on corporate greenwashing behavior. Columns (1) and (2) display the results from the model with time-fixed effects and the model with both time and individual fixed effects, respectively, without controlling for covariates. The results indicate that digital transformation significantly inhibits greenwashing behavior at a 1% level. In columns (3) and (4), we introduce control variables, and the results show that the regression coefficient for digital transformation remains significantly negative at a 1% level, indicating that digital transformation significantly suppresses corporate greenwashing behavior. From the perspective of control variables, enterprises with high cash flow have more funds to use for packaging their environmental image. Those with a high proportion of independent directors may, due to information asymmetry or insufficient motivation for supervision, instead reduce the restraint on greenwashing behaviors. In addition, this paper also conducts a series of robustness tests, including controlling for city-level control variables, using double machine learning models, replacing core variables, changing the sample, controlling for interaction fixed effects, and adopting higher-level clustering standard errors. The results of these tests indicate that the baseline regression results still hold. The specific details of the robustness tests can be found in the Appendix.

**Table 3:** Benchmark regression results.

	(1)	(2)	(3)	(4)
	*GW*	*GW*	*GW*	*GW*
*Dig*	−0.078^***^	−0.071^***^	−0.084^***^	−0.070^***^
	(0.022)	(0.020)	(0.022)	(0.020)
*Size*			0.136***	0.022
			(0.024)	(0.032)
*Lev*			−0.658***	−0.159
			(0.132)	(0.130)
*Fixed*			−0.065	−0.058
			(0.131)	(0.162)
*ListAge*			−0.121***	−0.005
			(0.030)	(0.058)
*Cashflow*			0.660***	0.283*
			(0.232)	(0.163)
*Board*			0.181	0.119
			(0.131)	(0.120)
*Dual*			0.109**	0.051
			(0.051)	(0.042)
*Indep*			0.008*	0.006*
			(0.004)	(0.003)
Time-fixed	Y	Y	Y	Y
City-fixed	N	Y	N	Y
Obs	13079	13079	13079	13079
R^2^	0.104	0.138	0.132	0.139

Note. *, **, and *** denote significance at the 10%, 5%, and 1% levels, with robust standard errors in parentheses, as in the following tables.

### 4.2. Addressing endogeneity concerns

Enterprises that value genuine environmental protection may also adopt digital transformation to improve the efficiency of environmental management. Therefore, there may be a bidirectional causal relationship between digital transformation and greenwashing behaviors. In addition, there are some key variables not included in the model that affect both enterprises’ digital transformation and greenwashing behaviors, which may also lead to endogeneity issues. To address this, we use lagged explanatory variables to mitigate the bidirectional causal relationship, and further adopt the Difference-in-Differences (DID) approach and instrumental variable method to alleviate endogeneity.

#### 4.2.1. Lagging explanatory variable.

To mitigate reverse causality, we lag the digital transformation variable by 1–4 periods respectively. As shown in columns (1) to (4) of Table 4, the coefficients of the digital transformation variable with first to fourth-order lags all exhibit a significant negative correlation with corporate greenwashing behavior, and pass the test at the 1% significance level. The impact coefficients of digital transformation with first to third-order lags are larger than those in the baseline regression results. This indicates that the direction of estimation bias potentially caused by reverse causality is downward—meaning the true impact coefficient is underestimated—thus verifying the robustness of the baseline regression results.

**Table 4:** Regression results of lagged explanatory variables and DID method.

	(1)	(2)	(3)	(4)	(5)
	*GW*	*GW*	*GW*	*GW*	*GW*
L.*Dig*	−0.098^***^				
	(0.019)				
L2.*Dig*		−0.096^***^			
		(0.020)			
L3.*Dig*			−0.087^***^		
			(0.020)		
L4.*Dig*				−0.057^***^	
				(0.020)	
*Broadband China pilot*	−1.055	−1.593^*^	−2.437^**^	−3.123^**^	−0.089^*^
	(0.838)	(0.968)	(1.114)	(1.311)	(0.051)
Control variables	Y	Y	Y	Y	Y
Time-fixed	Y	Y	Y	Y	Y
City-fixed	Y	Y	Y	Y	Y
Obs	11535	10196	8927	7720	12469
R^2^	0.146	0.156	0.164	0.159	0.134

#### 4.2.2. Difference-in-differences approach.

We also apply a difference-in-differences (DID) method to mitigate endogeneity. In 2013, the State Council of China introduced the Broadband China Strategy Implementation Plan. Subsequently, between 2014 and 2016, the Ministry of Industry and Information Technology and the National Development and Reform Commission selected 120 cities as pilot locations to promote the construction and expansion of information infrastructure. Recognizing that such infrastructure serves as a critical enabler of digital development, the Broadband China pilot policy is utilized as an external shock to measure the impact of digital transformation. The DID model results, presented in column (5) of Table 4, reveal that the policy significantly curbed greenwashing practices among corporations, offering robust evidence for the study’s conclusions. The results of the parallel trend test are shown in Figure A1 in the S1 Appendix.

#### 4.2.3. Instrumental variable approach.

To further alleviate the endogeneity of omitted explanatory variables, this study uses the instrumental variable approach. The 1984 postal and telecom data of corporations’ cities is initially used as an instrumental variable. Local historical telecom infrastructure impacts modern internet adoption, fulfilling the relevance condition, while not directly affecting greenwashing, meeting exogeneity. As this data is cross – sectional, following Yuan, Xiao [42], a time series variable is combined to form a panel instrumental variable, with the interaction between 1984 fixed-telephone-per-100-people in the city and the previous year’s national internet users as the first one (*IV1*). Secondly, based on Zhang, Yang [43], the interaction of the distance from the city to the centroid of “Eight Verticals and Eight Horizontals” fiber optic cities and a time trend is the second instrumental variable (*IV2*). Proximity to fiber optic cities aids digital transformation (relevance), and the network structure and distance are exogenous, not directly influencing greenwashing (exogeneity).

Table 5 presents the regression results using the two-stage least squares (2SLS) estimator based on IV1 in columns (1) and (2). From column (1), it can be observed that a higher number of fixed telephones and post offices in the city where the corporates are located in 1984 is associated with a higher degree of digital transformation among corporates, indicating a strong correlation between IV1 and digital transformation, without the presence of weak instrument problem (first-stage F-value of 11.600). Additionally, the Kleibergen-Paap rk LM statistic is 11.472, rejecting the null hypothesis of instrument weak identification at the 1% significance level. After addressing potential endogeneity concerns, the coefficient of digital transformation on corporates’ greenwashing behavior remains negative and significant at the 1% level. Columns (3) and (4) in Table 5 present the regression results using the 2SLS estimator based on IV2. These results further confirm the robustness of the main finding.

**Table 5:** Two-stage least squares regression results.

	(1)	(2)	(3)	(4)
	Fist stage	Second stage	Fist stage	Second stage
	*Dig*	*GW*	*Dig*	*GW*
*Dig*		−0.863^*^		−0.524^**^
		(0.470)		(0.238)
*IV1*	97.982^***^			
	(28.764)			
*IV2*			−0.002^***^	
			(0.001)	
Control variables	Y	Y	Y	Y
Time-fixed	Y	Y	Y	Y
City-fixed	Y	Y	Y	Y
Kleibergen-Paap rk LM	11.472^***^		44.020^***^	
Fist stage F value	11.600^***^		43.990^***^	
Obs	11886	11886	12462	12462

## 5. Mechanism test and heterogeneity analysis

### 5.1. Mechanism test

#### 5.1.1. Weakening greenwashing motivation.

To illustrate the mechanism through which digital transformation weakens greenwashing behavior, this study first examines the impact of digital transformation on green innovation efficiency. Green innovation efficiency (*GIE*), as measured by the ratio of green innovation output to innovation input, is evaluated based on the research conducted by Liu, Pan [44]. Due to the lack of data on green innovation input for listed companies, this study approximates it using annual R&D expenditure. Additionally, the green innovation output of listed companies is measured by the total number of applications for green invention patents, utility models, and design patents, with the addition of one and the subsequent application of natural logarithm. The green patent data is sourced from the China National Intellectual Property Administration. Columns (1) and (2) in Table 6 presents the regression results of digital transformation on green innovation efficiency. It can be observed that regardless of the inclusion of control variables, digital transformation significantly promotes corporates’ green innovation efficiency.

**Table 6:** Mechanism test for weakening greenwashing motivations.

	(1)	(2)	(3)	(4)	(5)	(6)
	*GIE*	*GIE*	*FC*	*FC*	*KZ*	*KZ*
*Dig*	0.002^*^	0.001^*^	−0.021^***^	−0.003^*^	0.158^***^	0.055^**^
	(0.001)	(0.001)	(0.002)	(0.002)	(0.047)	(0.028)
_Cons	0.022^***^	−0.007	0.441^***^	3.493^***^	0.927^***^	8.706^***^
	(0.003)	(0.038)	(0.006)	(0.113)	(0.080)	(1.045)
Control variables	N	Y	N	Y	N	Y
Time-fixed	Y	Y	Y	Y	Y	Y
City-fixed	Y	Y	Y	Y	Y	Y
Obs	9674	9674	12318	12318	12318	12318
R^2^	0.044	0.046	0.184	0.531	0.164	0.644

Secondly, according to theoretical analysis, corporates under financial constraints are more likely to engage in greenwashing behavior in order to obtain financing. This study employs the *FC* index (see the specific calculation formulas in Fazzari, Hubbard [45]) and the *KZ* index (see the specific calculation formulas in Kaplan and Zingales [46]) to measure corporates’ financial constraints. A higher *FC* index indicates more severe financial constraints, while a lower *KZ* index indicates more severe financial constraints. Columns (3) to (6) in Table 6 presents the regression results of digital transformation on financial constraints. It can be observed that regardless of the inclusion of control variables, digital transformation significantly reduces financial constraints. The two aspects together suggest that digital transformation weakens corporate greenwashing motivations by enhancing green innovation efficiency and reducing financial constraints, thereby reducing greenwashing behavior.

#### 5.1.2. Reducing greenwashing opportunities.

To examine whether digital transformation enhances corporates’ internal control capabilities and reduces the opportunity for greenwashing, this study draws on the research by Meng, Li [47] and adopts the Dibo Internal Control Index (*IC*) as a measure of corporates’ internal control capabilities. The Dibo Internal Control Index is derived from the Dibo Internal Control Index Database developed by the Dibo Big Data Research Center. It is constructed from the perspective of the five elements of corporate internal control, namely internal environment, risk assessment, control activities, information and communication, and internal supervision. The results in Table 7 indicate that digital transformation significantly improves the quality of internal controls, with statistical significance at the 5% level. Combining the findings with the theoretical analysis, this suggests that digital transformation reduces the opportunity for greenwashing by strengthening corporates’ internal control capabilities.

**Table 7:** Mechanism test for reducing greenwashing opportunities.

	(1)	(2)
	*IC*	*IC*
*Dig*	0.035^**^	0.030^**^
	(0.014)	(0.013)
_Cons	6.841^***^	2.326^***^
	(0.022)	(0.715)
Control variables	N	Y
Time-fixed	Y	Y
City-fixed	Y	Y
Obs	9840	9840
R^2^	0.017	0.043

#### 5.1.3. Increasing greenwashing exposure.

To examine the mechanism through which digital transformation increases the exposure to greenwashing, this study first investigates whether digital transformation reduces the information asymmetry between corporates and stakeholders. Drawing on the research by Song, Zhou [48], the liquidity ratio, non-liquidity ratio, and the first principal component of reversal indicators are used as proxy variables to measure information asymmetry (ASY1), where an increase in its value indicates a higher degree of information asymmetry. Additionally, to enhance robustness, information asymmetry is also measured using the first and second principal components (ASY2). The data are sourced from the CSMAR database, and the calculation process of principal component analysis is shown in the Appendix. The results in columns (1) and (2) of Table 8 demonstrate that, regardless of the inclusion of control variables, digital transformation significantly reduces information asymmetry. Moreover, across different measures of information asymmetry, the regression results in columns (3) and (4) of Table 8 consistently show negative coefficients that are statistically significant at the 1% level.

**Table 8:** Mechanism test for increasing greenwashing exposure.

	(1)	(2)	(3)	(4)	(5)	(6)
	*ASY1*	*ASY1*	*ASY2*	*ASY2*	*MS*	*MS*
*Dig*	−0.051^***^	−0.033^***^	−0.084^***^	−0.055^***^	0.076^***^	0.054^***^
	(0.009)	(0.007)	(0.017)	(0.013)	(0.018)	(0.015)
_Cons	−0.124^***^	5.628^***^	−0.138^***^	9.301^***^	6.841^***^	2.326^***^
	(0.012)	(0.298)	(0.020)	(0.524)	(0.022)	(0.715)
Control variables	N	Y	N	Y	N	Y
Time-fixed	Y	Y	Y	Y	Y	Y
City-fixed	Y	Y	Y	Y	Y	Y
Obs	12486	12486	12486	12486	9840	9840
R^2^	0.297	0.419	0.285	0.396	0.017	0.043

Furthermore, based on the theoretical analysis, increased media attention can also enhance the exposure of greenwashing. To test whether digital transformation raises media attention, this study follows the methodology of Yu, Wang [49], using the total number of online news articles mentioning firms throughout the year as a proxy for media attention (*MS*), the data is sourced from CNRDS. The indicator is log-transformed (with a value of 1 added) for regression analysis. Columns (5) and (6) of Table 8 present the regression results of digital transformation on media attention. The findings reveal that digital transformation has a significantly positive effect on media attention, regardless of whether control variables are included. The two aspects collectively indicate that digital transformation reduces information asymmetry and increases media attention, thereby enhancing the exposure of corporate greenwashing and ultimately reducing greenwashing behavior.

### 5.2. Heterogeneity analysis

#### 5.2.1. Types of digital transformation.

Table 9 presents the impact of five sub-factors of digital transformation, namely Artificial Intelligence (*AI*), Big Data (*BD*), Cloud Computing (*Cloud*), Blockchain (*Block*), and Technological Application (*Apply*), on corporates’ greenwashing behavior. It is observed that the four underlying technologies in digital transformation have a significant negative effect on corporates’ greenwashing behavior at the 1% level. However, the impact of technological application is not significant, possibly because it solely views technology as a tool to improve efficiency and reduce costs, without effectively integrating environmental goals. Moreover, in terms of the magnitude of the estimated coefficients, blockchain and artificial intelligence have the most significant impact.

**Table 9:** Heterogeneity test for types of digital transformation.

	(1)	(2)	(3)	(4)	(5)
	*GW*	*GW*	*GW*	*GW*	*GW*
*IT*	−0.305^***^				
	(0.053)				
*BD*		−0.117^*^			
		(0.071)			
*Cloud*			−0.211^***^		
			(0.066)		
*Block*				−0.441^***^	
				(0.145)	
*Apply*					0.012
					(0.031)
_Cons	−1.086	−0.952	−0.973	−0.912	−0.864
	(0.720)	(0.721)	(0.728)	(0.723)	(0.727)
Control variables	Y	Y	Y	Y	Y
Time-fixed	Y	Y	Y	Y	Y
City-fixed	Y	Y	Y	Y	Y
Obs	13079	13079	13079	13079	13079
R^2^	0.141	0.139	0.137	0.138	0.136

#### 5.2.2. Micro-level: Firm characteristics.

First, following the approach of Yu, Zhao [50], we measure the short-termism of corporate management by the ratio of current short-term investments to the total assets at the beginning of the period, and perform a grouped regression analysis. According to the regression results in column (1) and (2) of Table 10, digital transformation significantly inhibits greenwashing behavior in firms with lower short-termism, while no significant effect is observed in firms with higher short-termism. Farsighted companies are more effective in using digital technologies to improve environmental measures and reduce greenwashing, while myopic companies, focused on short-term gains, may not adopt digital transformation effectively to enhance their environmental performance.

**Table 10:** Heterogeneity test for micro-level.

	(1)	(2)	(3)	(4)	(5)
	*GW*	*GW*	*GW*	*GW*	*GW*
	Lower short-termism	Higher short-termism	Growth stage	Maturity stage	Decline stage
*Dig*	−0.180^**^	−0.048^**^	−0.060^**^	−0.112^***^	0.012
	(0.084)	(0.021)	(0.026)	(0.034)	(0.036)
_Cons	−1.567	−1.770^**^	−1.143	−0.051	0.341
	(1.514)	(0.834)	(0.963)	(1.318)	(1.930)
Control variables	Y	Y	Y	Y	Y
Time-fixed	Y	Y	Y	Y	Y
City-fixed	Y	Y	Y	Y	Y
Obs	4519	8560	5798	5190	2071
R^2^	0.236	0.101	0.145	0.153	0.124

Secondly, using the cash flow classification method proposed by Dickinson [51], this study divides the lifecycle of corporates into five stages as outlined by Gort and Klepper [52]: introduction, growth, maturity, decline, and obsolescence. For analytical convenience, this study combines the introduction and growth stages into the “growth stage,” and the decline and obsolescence stages into the “decline stage.” As presented in columns (3) – (5) of Table 10, digital transformation has a significant negative impact on the greenwashing behavior of both growth stage and maturity stage companies. However, the impact of digital transformation on decline stage companies is not significant. This may be attributed to the greater operational pressures and resource constraints faced by these companies, making it difficult for them to effectively implement environmental measures during the transformation process.

#### 5.2.3. Macro-level: External environment.

First, this study measures the environmental regulatory efforts of local governments using the number of environmental enforcement cases. The sample is divided into high regulatory intensity and low regulatory intensity groups based on the median values of this indicators, and regression analysis is conducted accordingly. The number of environmental enforcement cases for each city is obtained from the Peking University Law Information database, as documented by Jiahao, Wenyu [53]. Columns (1) and (2) of Table 11 present the regression results based on the grouping of environmental enforcement cases. It is observed that the effect of digital transformation in suppressing corporates’ greenwashing behavior is significantly stronger in areas with weaker environmental regulatory intensity compared to areas with stronger regulatory intensity. This may be because in areas with weak regulatory enforcement, there are often issues of a shortage of human and material resources, making it difficult to conduct comprehensive and frequent on-site inspections of enterprises.

**Table 11:** Heterogeneity test for external environment.

	(1)	(2)	(3)	(4)
	*GW*	*GW*	GW	GW
	High regulatory intensity	Low regulatory intensity	High environmental concern	Low environmental concern
*Dig*	−0.049^*^	−0.129^***^	−0.047*	−0.074
	(0.026)	(0.031)	(0.028)	(0.045)
_Cons	−1.486	1.731^*^	−1.962	−2.566**
	(1.121)	(1.011)	(1.275)	(1.062)
Control variables	Y	Y	Y	Y
Time-fixed	Y	Y	Y	Y
City-fixed	Y	Y	Y	Y
Obs	8140	4939	5539	5446
R^2^	0.145	0.042	0.135	0.153

Second, this study uses Baidu Search Index to measure the public’s environmental awareness in different cities, reflecting the intensity of public opinion. Specifically, we compiled Baidu search data related to environmental topics from both PC and mobile platforms in each city, covering keywords such as “new energy,” “carbon,” “green,” “sustainability,” “emissions reduction,” “environmental protection,” “carbon dioxide,” “low carbon,” and “environmental awareness,” to accurately capture residents’ attention to environmental issues. As shown in column (3) and (4) of Table 11, when residents have a higher level of environmental awareness, digital transformation significantly reduces corporate greenwashing behavior. However, when residents have lower environmental awareness, the impact of digital transformation on greenwashing behavior is not significant. This suggests that in areas with high public opinion pressure, companies are more motivated to embrace digital transformation to enhance transparency and accountability, thereby reducing greenwashing behavior to protect their social reputation and public image.

## 6. Further discussion

### 6.1. Threshold effects of digital transformation

The impact of digital transformation on greenwashing behavior may vary at different stages. This section examines the threshold effect of digital transformation on corporate greenwashing and explores how different stages of transformation affect such behavior. We set the trimming proportion to 0.02 and the number of bootstrap replications to 50. Table 12 presents the existence test for thresholds in an unbalanced panel. After repeated sampling with 300 iterations, the results show that the single and double thresholds are significant, while the triple threshold is not. This indicates a double-threshold effect of digital transformation on corporate greenwashing. Based on the test results, digital transformation levels below 0.727 are categorized as the initial stage, levels between 0.727 and 5.218 as the intermediate stage, and levels above 5.218 as the mature stage.

**Table 12:** Threshold significance test.

Threshold count	Threshold value	RSS	MSE	Fstat	P value	10% critical value	5% critical value	1% critical value
1	5.218	2094.975	0.664	13.27	0.060**	10.437	13.396	16.414
2	0.727	2088.970	0.662	10.59	0.060**	8.397	11.344	11.772
3	0.119	2082.392	0.660	8.45	0.660	13.320	17.895	21.318

Table 13 presents the results of the threshold regression analysis for an unbalanced panel, highlighting the distinct stage-specific effects of digital transformation on corporate greenwashing. In the initial stage of digital transformation, no significant impact on greenwashing behavior is observed. This is likely because firms in this phase often channel most resources into testing digital tools (such as basic data management software) and training employees on new systems, leaving little capacity to link digitalization to environmental oversight or greenwashing prevention. However, once digital transformation surpasses the threshold of 0.727 and enters the intermediate stage, the coefficient becomes −0.088 and is significant at the 1% level. This may be attributed to companies primarily focusing on exploring and investing in digital technologies during the initial stage, while still adapting to new processes and cultures. As transformation progresses into the intermediate stage, firms are better equipped to leverage digital technologies—like real-time environmental data tracking platforms—to enhance transparency; this not only reduces information asymmetry between firms and stakeholders but also makes deceptive green claims harder to conceal, leading to a more significant suppression of greenwashing. When digital transformation exceeds the second threshold of 5.218 and enters the mature stage, its suppressive effect on greenwashing further strengthens, with the coefficient reaching −0.105, also significant at the 1% level. By this stage, digitalization is fully integrated into core operations (e.g., supply chain carbon accounting or ESG digital reporting), allowing firms to automate environmental monitoring and reduce human errors or intentional data manipulation that often enable greenwashing.

**Table 13:** Threshold estimation results.

	(1)
	*GW*
*Dig* < 0.727	0.080
	(0.105)
0.727 < *Dig* < 5.218	−0.088^***^
	(0.034)
*Dig* > 5.218	−0.105^***^
	(0.021)
_Cons	−4.049^***^
	(0.701)
Control variables	Y
Time-fixed	Y
City-fixed	Y
Obs	4325
R^2^	0.083

### 6.2. Economic consequence analysis

The empirical results above indicate that digital transformation effectively suppresses corporate greenwashing. However, greenwashing often stems from motives such as reducing green investment costs or attracting green financing. This raises a critical question: does digital transformation, while curbing greenwashing, negatively impact a firm’s financial performance? Furthermore, can the advancement of digital transformation enable firms to balance green responsibility with long-term development? To address these questions, this study further examines the impact of digital transformation on corporate operations and growth while suppressing greenwashing behavior.

This study evaluates corporate sustainability using total factor productivity (TFP). TFP is calculated using both the OP and LP methods, denoted as *TFP_OP* and *TFP_LP* (Levinsohn & Petrin, 2003; Olley & Pakes, 1996). Meanwhile, corporate financial performance is assessed using key indicators, including sales revenue (*Sale*), return on equity (*ROE*), return on assets (*ROA*), and net profit growth rate (*NetProfitGrowth*).

Table 14 shows that the interaction terms between digital transformation and greenwashing are not significant for all dependent variables. This indicates that while digital transformation suppresses greenwashing, it does not negatively impact corporate sustainability or financial performance. The possible reasons are twofold. First, digital transformation enhances transparency and efficiency, reducing the incentive for greenwashing. Technologies like IoT, big data, and blockchain enable real-time monitoring and accurate reporting of environmental impacts, optimizing resource allocation and cutting unnecessary costs. This offsets any financial losses from reduced greenwashing-related gains. Second, digital transformation helps firms better identify and manage environmental risks, develop effective green strategies, and comply with regulations, reducing penalties and enhancing reputation. This boosts brand value, attracting consumers and investors while supporting financial performance.

**Table 14:** Economic consequences test.

	(1)	(2)	(3)	(4)	(5)	(6)
	*TFP_OP*	*TFP_LP*	*Sale*	*ROE*	*ROA*	*NetProfitGrowth*
*Dig*	0.006	0.009	0.008	−0.001	−0.002^*^	−0.050
	(0.011)	(0.011)	(0.011)	(0.002)	(0.001)	(0.046)
*GW*	0.028^***^	0.020^***^	0.014^***^	0.005^***^	0.002^***^	0.147^***^
	(0.005)	(0.005)	(0.005)	(0.001)	(0.001)	(0.043)
*Dig × GW*	−0.001	0.002	0.003	−0.001	−0.001	−0.014
	(0.003)	(0.003)	(0.002)	(0.001)	(0.000)	(0.016)
_Cons	−1.968^***^	−3.421^***^	1.455^**^	−0.190^**^	−0.017	0.487
	(0.439)	(0.433)	(0.594)	(0.095)	(0.035)	(2.228)
Control variables	Y	Y	Y	Y	Y	Y
Time-fixed	Y	Y	Y	Y	Y	Y
City-fixed	Y	Y	Y	Y	Y	Y
Obs	12505	12505	13079	12854	12854	12854
R^2^	0.452	0.572	0.744	0.148	0.230	0.028

## 7. Conclusions and implications

### 7.1. Conclusions

This study examines how digital transformation impacts corporate greenwashing. Using the fraud risk factor theory, it devises a framework to understand greenwashing motives and proposes three ways digital transformation curbs greenwashing: weakening motives, reducing opportunities, and increasing exposure. Empirically, with panel data from 2009–2022 of listed firms and a two – way fixed effects model, plus robustness and endogeneity checks, digital transformation is found to significantly inhibit greenwashing. Further analysis shows it mitigates motives via green innovation and financing, cuts opportunities through better internal control, and increases exposure by reducing information asymmetry. Heterogeneity analyses show blockchain and AI have strong effects, especially for growth/mature, non – myopic firms. A double-threshold effect exists, with the suppressive impact strengthening in intermediate and mature digital stages, and digital transformation reduces greenwashing without harming corporate sustainability or finances.

### 7.2. Policy implications

Based on the study’s findings, there are practical and policy implications for corporate management and policymakers.

(1) Promoting digital technologies for green governance. The findings indicate that digital transformation can effectively curb greenwashing by enhancing green innovation, improving financing channels, strengthening internal control, and reducing information asymmetry. Firms should actively adopt technologies such as blockchain, artificial intelligence, and big data to build traceable and verifiable environmental disclosure systems. Policymakers may provide tax incentives, green credit, and technological subsidies to encourage firms to apply these digital tools, thereby weakening the motives and opportunities for greenwashing at the source.
(2) Building digital regulatory and market-based constraint mechanisms. Results suggest that digitalization significantly increases the likelihood of exposing greenwashing, with stronger effects in the growth and maturity stages. Governments should accelerate the establishment of environmental regulatory platforms based on big data and blockchain, enabling dynamic cross-checking of corporate environmental disclosures with third-party monitoring data. At the same time, industry associations, media, and investors can leverage digital tools to strengthen supervision, fostering a transparent environment for green development.
(3) Focusing on differentiated support and long-term sustainability. Heterogeneity analysis shows that the impacts of digital transformation vary by firm characteristics and digitalization stage, with small and medium-sized enterprises (SMEs) facing greater barriers to digital adoption. Governments should provide targeted financial support, technical training, and demonstration projects to help SMEs overcome digital thresholds. Moreover, since digital transformation does not undermine firms’ financial performance, both enterprises and policymakers can regard it as a long-term strategic tool to achieve a “win–win” outcome of green and economic performance.

### 7.3. Limitations and future research

This study has certain limitations. First, the measurement of digital transformation is based on keyword frequency in annual reports, which may not fully reflect the depth and quality of firms’ digitalization. Future research could employ more direct indicators, such as digital infrastructure investment or patent data, to enhance measurement accuracy. Second, the analysis is limited to Chinese listed firms, and the findings may vary under different institutional and regulatory contexts. Comparative studies across countries or regions would help to improve the external validity and generalizability of the conclusions.

## Supporting information

S1 Appendix Supplementary material. (DOCX)

S1 Data Data. (XLSX)
