# Supplementary material for: How digital transformation curb greenwashing: Insights from fraud risk factor theory
Source: PLoS One. 2026 Feb 2;21(2):e0339282. doi: 10.1371/journal.pone.0339282 (PMC12863495; doi:10.1371/journal.pone.0339282)
Supplement: S1 Appendix — (DOCX) [file pone.0339282.s001.docx]

## 1. Measurement of digital transformation

This study employs Python web scraping technology to extract keywords related to digital transformation from the annual reports of listed companies. These keywords are sourced from Chinese local government reports, the 14th Five-Year Plan for Digital Economy Development, and research reports on the digital transformation of Chinese enterprises released by authoritative institutions such as Tsinghua University.

In line with the conceptual definition of digital transformation in this paper, the keywords of digital transformation are divided into two levels: "underlying technical architecture" and "technical practical application". Among them, "underlying technical architecture" includes four major digital technologies, namely artificial intelligence, big data, cloud computing, and blockchain; "technical practical application" emphasizes the application and transformation of digital technologies in actual business scenarios. In total, there are more than 100 keywords related to digital transformation, and the specific keywords are shown in Table A1.

Subsequently, the frequency of these characteristic words is used to measure the digitalization degree of enterprises. The frequency of words related to digital transformation mentioned in enterprises' annual reports serves as the measurement indicator for the degree of digitalization. Given the potential significant differences in the volume of annual report texts disclosed by different enterprises, this study refers to the processing method of Yuan Chun et al. (2021): the word frequency related to digital transformation is divided by the total word frequency of the annual report text, and finally the corporate digital transformation index (Dig) is obtained. For the convenience of expression, this indicator is multiplied by 10,000 in this paper.

Before 2009, enterprises' understanding of digitalization was generally limited to specific technologies or systems, lacking an overall understanding and strategic acceptance of digitalization. It was not until after 2009 that, with the rapid development and popularization of Internet technology, Chinese enterprises began to gradually recognize the importance of digital transformation and started to adopt digital tools and platforms on a large scale. In addition, since the Huazheng ESG rating data has included environmental dimension information starting from 2009, the available greenwashing data begins in 2009. Based on the above reasons, the sample period of this study is determined to be from 2009 to 2022.

Table A1 Key words List of Digital transformation

| Underlying Technical Architecture | Artificial Intelligence (AI) | Artificial intelligence, business intelligence, image understanding, investment decision support, intelligent data analysis, intelligent robots, machine learning, deep learning, semantic search, biometrics, face recognition, speech recognition, identity verification, autonomous driving, natural language processing |
| --- | --- | --- |
|  | Big Data | Big data, data mining, text mining, data visualization, heterogeneous data, credit, augmented reality (AR), mixed reality (MR), virtual reality (VR) |
|  | Cloud Computing | Cloud computing, stream computing, graph computing, in-memory computing, secure multi-party computation (SMPC), brain-inspired computing, green computing, cognitive computing, converged architecture, 100-million-level concurrency, EB-level storage, Internet of Things (IoT), cyber-physical systems (CPS) |
|  | Blockchain | Blockchain, digital currency, distributed computing, differential privacy technology, smart financial contracts |
| Technical Practical Application | Mobile Internet, Industrial Internet, mobile Internet, Internet-based healthcare, e-commerce, mobile payment, third-party payment, NFC payment, smart energy, B2B, B2C, C2B, C2C, O2O, Internet interconnection, smart wearables, smart agriculture, smart transportation, smart healthcare, intelligent customer service, smart home, intelligent investment advisory, smart cultural tourism, smart environmental protection, smart grid, smart marketing, digital marketing, unattended retail, Internet finance, digital finance, financial technology (FinTech), financial technology (FinTech), quantitative finance, open banking | |

## 2. Robustness test

### 2.1. Controlling for city-level control variables

Considering that greenwashing behavior may be influenced by macroeconomic characteristics across different regions, this study further incorporates city-level economic development, population density, technological level, and human capital as control variables to test the robustness. Specifically, the level of economic development is measured using the logarithm of per capita regional GDP; population density is quantified by the logarithm of the number of people per square kilometer; technological level is assessed by the ratio of research and development expenditures to regional GDP; and human capital is measured by the logarithm of the number of university students per 10000 people. As shown in Table A2, after adding these regional control variables, the coefficient of digital transformation on greenwashing remains significantly negative at the 1% level, indicating that the baseline results are robust.

Table A2 Regression results after adding city-level control variables

|  | (1) | (2) | (3) |
| --- | --- | --- | --- |
|  | *GW* | *GW* | *GW* |
| *Dig* | -0.069^***^ | -0.086^***^ | -0.070^***^ |
|  | （0.022） | （0.023） | （0.020） |
| _Cons | -6.051^***^ | -2.166^***^ | -0.792 |
|  | （0.648） | （0.743） | （1.185） |
| Control variables | Y | Y | Y |
| Time-fixed | N | Y | Y |
| City-fixed | N | N | Y |
| Obs | 12492 | 12492 | 12492 |
| R^2^ | 0.062 | 0.133 | 0.136 |

### 2.2. Double machine learning models

Linear regression models assume a straightforward linear relationship between control variables, digital transformation, and greenwashing. This assumption may not adequately capture true confounding factors, potentially leading to model specification bias. Double Machine Learning (DML), utilizing advanced machine learning algorithms, offers significant advantages in modeling nonlinear relationships and effectively addresses estimation bias caused by model misspecification (Chernozhukov et al., 2018). Additionally, DML mitigates regularization bias found in traditional machine learning approaches, ensuring the unbiasedness of coefficient estimates for explanatory variables even in small samples. Therefore, this study employs DML to perform a robustness check on the relationship between digital transformation and corporate greenwashing behavior.

The results from the DML model based on the Random Forest algorithm are presented in column (1) of Table A2. It is evident that the coefficient for digital transformation remains significantly negative at the 1% level. To ensure the robustness, the Random Forest algorithm is replaced sequentially with Lasso regression and Gradient Boosting for re-estimation. The findings, as displayed in columns (2) and (3) of Table A2, confirm that the results are robust to changes in the machine learning algorithms used. Additionally, the sample split ratio is varied from 1:4 to 1:2 and 1:7 to examine the potential impact of different sample split ratios on this study's conclusions. As indicated in columns (4) and (5) of Table A3, the suppressive effect of digital transformation on greenwashing behavior remains significant, further affirming the robustness of the baseline regression results.

Table A3 DML model estimation results

|  | (1) | (2) | (3) | (4) | (5) |
| --- | --- | --- | --- | --- | --- |
|  | GW | GW | GW | GW | GW |
| Dig | -0.062*** | -0.037** | -0.034*** | -0.062*** | -0.056*** |
|  | （0.016） | （0.015） | （0.015） | （0.017） | （0.017） |
| Control variables | Y | Y | Y | Y | Y |
| Time-fixed | Y | Y | Y | Y | Y |
| City-fixed | Y | Y | Y | Y | Y |
| Obs | 13079 | 13079 | 13079 | 13079 | 13079 |
| Algorithms | Random forest | Lasso | Gradient boosting | Random forest | Random forest |
| Sample split ratio | 1：4 | 1：4 | 1：4 | 1：2 | 1：7 |

### 2.3. Replacement of core variables

To mitigate estimation bias caused by measurement errors in the dependent variable, a robustness check is conducted by substituting the dependent variable. We use the environmental responsibility scores from the Corporate Social Responsibility Index published by Hexun as a measure of corporate environmental performance. The degree of corporate greenwashing (*GW2*) is recalculated. The results of substituting the dependent variable are presented in columns (1) and (2) of Table A4. It is evident that digital transformation continues to have a significant inhibitory effect on greenwashing behavior, indicating that the regression results support the conclusions of the baseline regression even after substituting the dependent variable.

In addition, this study adopts the approach of Yuan et al. (2021) to construct a lexicon of corporate digitalization terms based on the semantic framework of national policies. Machine learning models are used to analyze the text of listed companies' annual reports, statistically calculating the proportion of digitization-related word frequencies to the total word frequencies. This new indicator of corporate digital transformation (*Digital_index*) is then constructed to rerun the regression. The regression results are shown in columns (3) and (4) of Table A4, regardless of whether control variables are added or not, the impact of digital transformation on corporate greenwash behavior is significantly negative, and all are significant at the 1% level.

Table A4 Results after replacing core variables

|  | (1) | (2) | (3) | (4) |
| --- | --- | --- | --- | --- |
|  | *GW2* | *GW2* | *GW* | *GW* |
| *Dig* | -0.132^***^ | -0.105^***^ |  |  |
|  | （0.023） | （0.024） |  |  |
| *Digital_index* |  |  | -0.317*** | -0.315*** |
|  |  |  | （0.112） | （0.113） |
| _Cons | -2.021^***^ | 3.637^***^ | -0.219*** | -1.084 |
|  | （0.059） | （1.384） | （0.035） | （0.732） |
| Control variables | N | Y | N | Y |
| Time-fixed | Y | Y | Y | Y |
| City-fixed | Y | Y | Y | Y |
| Obs | 10663 | 10663 | 13079 | 13079 |
| R^2^ | 0.439 | 0.443 | 0.137 | 0.138 |

### 2.4. Other robustness test

First, Replacement sample. Given the significant uniqueness of municipalities compared to prefecture-level cities in terms of institutional structure and economic development trajectory, this study excludes corporates in China's four municipalities, namely Beijing, Tianjin, Shanghai, and Chongqing, and conducts regression analysis again. The regression results are shown in Column (1) of Table A5. Additionally, due to the impact of the COVID-19 epidemic at the end of 2019, the data for listed companies after 2020 may fluctuate significantly, thereby affecting the regression results. Therefore, we remove the samples from 2020 to 2022 and conducts regression analysis again. The regression results are shown in Column (2) of Table A5. Second, Controlling for interaction fixed effects. In order to control for the time-varying characteristics of cities and industries, this study incorporates fixed effects of the interaction of cities, industries, and years into the model. The regression results are shown in Columns (3) to (4) of Table A5. Third, Higher-level clustering standard error. While the standard errors in the baseline regression are clustered at the firm level, we further clusters the standard errors at the municipal and provincial levels. The regression results are shown in Columns (5) to (6) of Table A5. It can be observed that, after conducting a series of robustness tests, the coefficients remain significantly negative.

Table A5 Other robustness test

|  | (1) | (2) | (3) | (4) | (5) | (6) |
| --- | --- | --- | --- | --- | --- | --- |
|  | GW | GW | GW | GW | GW | GW |
| Dig | -0.069*** | -0.081*** | -0.067*** | -0.036* | -0.070*** | -0.070*** |
|  | （0.026） | （0.013） | （0.022） | （0.019） | （0.020） | （0.017） |
| _Cons | -0.264*** | 1.233** | -1.080 | -0.927 | -0.873 | -0.873 |
|  | （0.041） | （0.533） | （0.936） | （0.738） | （0.734） | （0.882） |
| Control variables | Y | Y | Y | Y | Y | Y |
| Time-fixed | Y | Y | Y | Y | Y | Y |
| City-fixed | Y | Y | Y | Y | Y | Y |
| Obs | 9253 | 10087 | 13079 | 13079 | 13079 | 13079 |
| R2 | 0.151 | 0.040 | 0.662 | 0.638 | 0.589 | 0.589 |

## 3. Parallel trend test

The application of the DID method requires the fulfillment of the parallel trend assumption. Following the approach in previous literature, this study excludes the pre-implementation period as the base period for conducting a parallel trends test. Given the limited sample size for the period five years before policy implementation, this study combines it with the -5th period. Additionally, recent econometric literature suggests that the estimates of two-way fixed effects (TWFE) in a DID model are actually weighted averages of treatment effects across different time periods and may involve negative weights (Callaway & Sant’Anna, 2021; De Chaisemartin & d'Haultfoeuille, 2024). In cases where negative weights are present, the weighted average treatment effect obtained may be in the opposite direction of the true treatment effect. Therefore, using TWFE estimates for parallel trends testing may introduce bias. To address this issue, Sun and Abraham (2021) propose the use of intersection weights (IW) estimates, which weight treatment effects based on the sample sizes across different treatment periods. This approach avoids the problem of negative weights and is applicable to the unbalanced panel data used in this study. Hence, IW estimates are employed as robustness measures in this study. Figure A1 displays the results of the parallel trends test using both estimators, indicating similar findings. The treatment and control groups demonstrate similar pre-treatment trends, providing evidence that there is no pre-existing trend, thus passing the parallel trends test in this study sample.


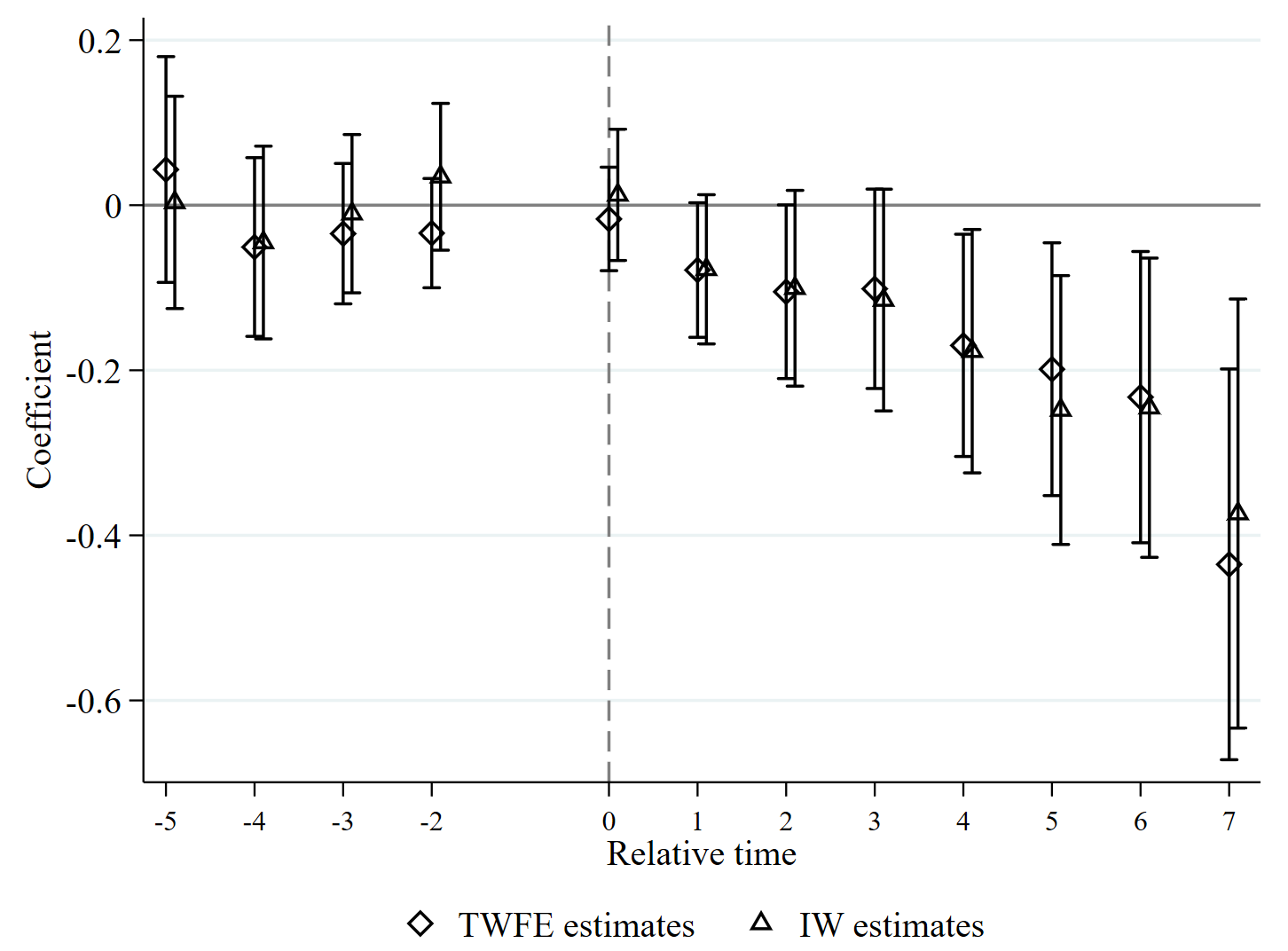


Figure A1 Parallel trend test for the Broadband China pilot

## 4. The calculation process of principal component analysis

The core of Principal Component Analysis (PCA) is to project multivariate data onto new orthogonal dimensions (principal components) through linear transformation. Its key calculation formulas revolve around covariance matrix solution and eigenvalue decomposition, mainly divided into 4 steps.

### 4.1. Data Standardization

There are large differences in the dimensions of original variables, standardization is required first to eliminate their influence. The formula is:

Standardized variable: $z_{ij}=\frac{x_{ij}-\bar{x}_{j}}{s_{j}}$

Where $x_{ij}$ is the $j$-th original variable of the $i$-th sample, $\bar{x}_{j}$ is the mean of the $j$-th variable, and $s_{j}$ is the standard deviation of the $j$-th variable.

### 4.2. Calculation of the Covariance Matrix

The covariance matrix reflects the degree of linear correlation between variables and is the core input of PCA. The formula is:

Covariance matrix$C$(dimension$p\times p$, where $p$ is the number of original variables): $(C_{jk}=\frac{1}{n-1}\sum_{i=1}^{n} (z_{ij}-\bar{z}_{j})(z_{ik}-\bar{z}_{k}))$

Where $C_{jk}$ is the covariance between the $j$-th and $k$-th standardized variables, and $n$ is the number of samples. After standardization, $\bar{z}_{j}=0$, so the formula can be simplified to $C_{jk}=\frac{1}{n-1}\sum_{i=1}^{n} z_{ij}z_{ik}$.

### 4.3. Solution of Eigenvalues and Eigenvectors

Eigenvalue decomposition is performed on the covariance matrix to obtain the "direction" (eigenvector) and "explanatory power" (eigenvalue) of the principal components. The formula is:

Eigenvalue equation: $C\mathbf{u}=\lambda\mathbf{u}$

Where $\lambda$ is the eigenvalue of the covariance matrix $C$ (sorted in descending order: $\lambda_{1}\geq\lambda_{2}\geq...\geq\lambda_{p}$), and $\mathbf{u}$ is the eigenvector corresponding to $\lambda$ (a unit vector, i.e., $\mathbf{u}^{T}\mathbf{u}=1$).

### 4.4. Calculation of Principal Component Scores

The standardized data is projected onto the eigenvectors to obtain the scores of each sample on the principal components. The formula is:

Score of the m-th principal component $F_{im}:F_{im}=\sum_{j=1}^{p} z_{ij}u_{jm}$

Where $u\_\left\{jm\right\}$ is the loading of the $j$-th variable on the $m$-th principal component (i.e., the $j$-th element of the eigenvector $\mathbf{u}_{m}$, and $F\_\left\{im\right\}$ represents the value of the $i$-th sample on the $m$-th principal component.

**References**

Callaway, B., & Sant’Anna, P. H. (2021). Difference-in-differences with multiple time periods. *Journal of Econometrics*, *225*(2), 200-230.

Chernozhukov, V., Chetverikov, D., Demirer, M., Duflo, E., Hansen, C., Newey, W., & Robins, J. (2018). Double/debiased machine learning for treatment and structural parameters. In: Oxford University Press Oxford, UK.

De Chaisemartin, C., & d'Haultfoeuille, X. (2024). Difference-in-differences estimators of intertemporal treatment effects. *Review of Economics and Statistics*, 1-45.

Sun, L., & Abraham, S. (2021). Estimating dynamic treatment effects in event studies with heterogeneous treatment effects. *Journal of Econometrics*, *225*(2), 175-199.

Yuan, C., Xiao, T., Geng, C., & Sheng, Y. (2021). Digital transformation and division of labor between enterprises: Vertical specialization or vertical integration. *China industrial economics*, *9*, 137-155.
